# The Role of the Axillary Artery as a Second Access Choice in TAVI Procedures

**DOI:** 10.21470/1678-9741-2020-0343

**Published:** 2021

**Authors:** Ana Paula Tagliari, Rodrigo Petersen Saadi, Enrico Ferrari, Maurizio Taramasso, Eduardo Keller Saadi

**Affiliations:** 1 Department of Cardiac Surgery, University Hospital of Zurich, University Heart Center Zurich, Zurich, Switzerland.; 2 Department of Cardiovascular Surgery, Hospital São Lucas da Pontifícia Universidade Católica do Rio Grande do Sul (PUCRS), Porto Alegre, Rio Grande do Sul, Brazil.; 3 Postgraduate Program in Cardiology and Cardiovascular Sciences, Universidade Federal do Rio Grande do Sul (UFRGS), Porto Alegre, Rio Grande do Sul, Brazil.; 4 Department of Cardiovascular Surgery, Hospital de Clínicas de Porto Alegre (HCPA), Porto Alegre, Rio Grande do Sul, Brazil.; 5 Department of Cardiac Surgery, Cardiocentro Ticino, Lugano, Switzerland.

**Keywords:** Aortic Valve Stenosis, Transcatheter Aortic Valve Implantation, Axillary Artery

## Abstract

With transcatheter aortic valve implantation (TAVI) technology expanding its indications for low-risk patients, the number of TAVI-eligible patients will globally grow, requiring a better understanding about the second-best access choice. Regarding the potential access sites, the transfemoral retrograde route is recognized as the standard approach and first choice according to current guidelines. However, this approach is not suitable in up to 10-15% of patients, for whom an alternative non-femoral access is required. Among the alternative non-femoral routes, the transaxillary approach has received increasing recognition due to its proximity and relatively straight course from the axillary artery to the aortic annulus, which provides a more accurate device deployment. Here we discuss some particular aspects of the transaxillary access, either percutaneously performed or by cutdown dissection.

**Table:** 

Abbreviations, acronyms & symbols	
**AS**	**= Aortic stenosis**
**CI**	**= Confidence interval**
**EuroScore I**	**= European System for Cardiac Operative Risk Evaluation I**
**LIMA**	**= Left internal mammary artery**
**OR**	**= Odds ratio**
**STS**	**= Society of Thoracic Surgeons**
**TAVI**	**= Transcatheter aortic valve implantation**
**TAx**	**= Transaxillary**
**TF**	**= Transfemoral**

## INTRODUCTION

Transcatheter aortic valve implantation (TAVI) is a well-established treatment option for patients with severe symptomatic aortic stenosis (AS) regardless of the risk class, but especially in those who are inoperable or at high surgical risk.

Among the potential access sites, the transfemoral (TF) retrograde route is recognized as the standard approach and first choice according to current guidelines^[1,2]^. At the beginning of TAVI experience, however, alternative non-TF accesses were needed in up to 30% of patients^[3]^, and even now, they are still indicated in 10-15% of the procedures^[4,5]^.

**Fig. 1:**
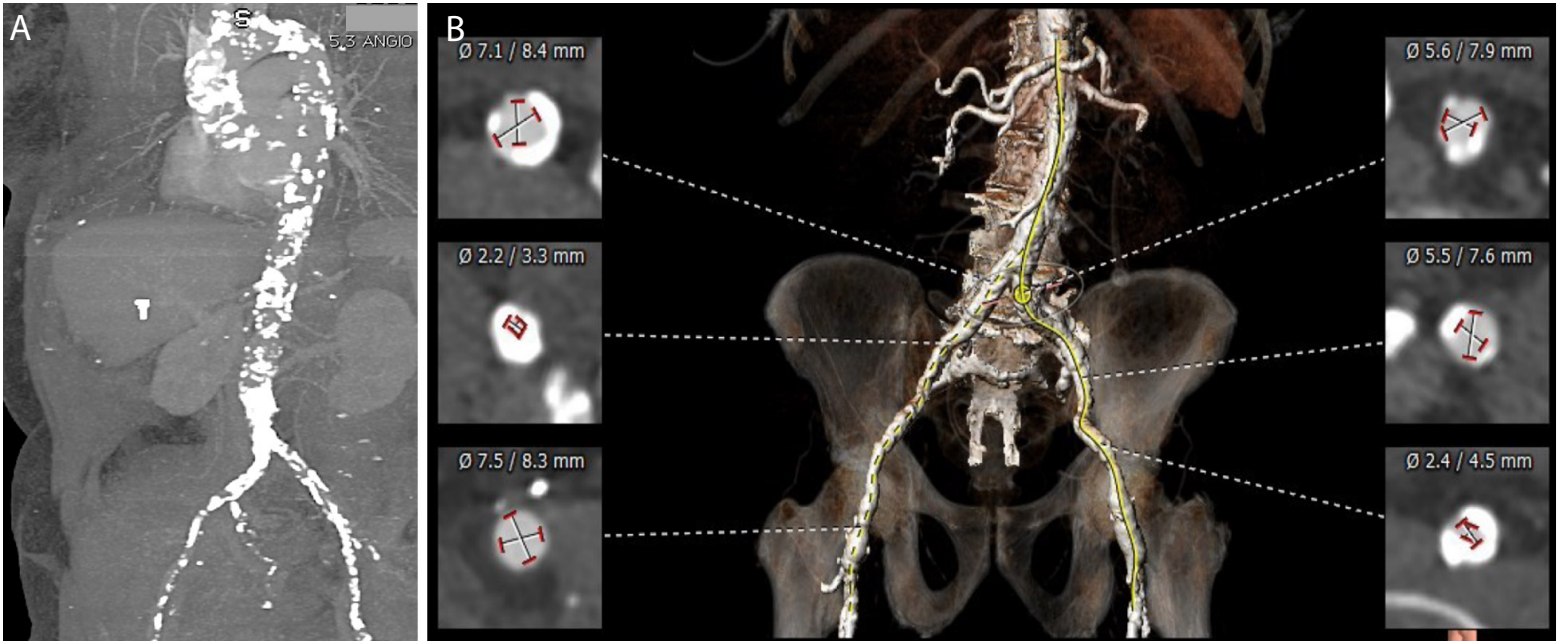
Computed tomography angiography reconstruction showing extensive aorta calcification (A) and important peripheral artery disease with circumferential calcification precluding transfemoral access (B).

Hostile aorto-iliofemoral anatomy due to extensive calcification, atherosclerosis, tortuosity, small vessel diameter, or presence of previously implanted arterial grafts represent the main reasons for TF access preclusion^[4]^ (Figure 1). In this setting, transapical, direct aortic, transcarotid, transcaval, and trans-subclavian/transaxillary (TAx) are possible alternative routes. Among these options, the TAx approach has received increasing recognition and has currently been used in up to 20% of TAVI procedures in some centers^[6,7]^.

One of the TAx advantages is the proximity and relatively straight course from the axillary artery to the aortic annulus, which provides a more accurate device deployment^[8]^. Despite this theoretical benefit, the lack of randomized clinical trials and evidence-based guideline recommendations make the TAx role, as the second route of choice, to be questionable.

Here we discuss some particular aspects of the TAx access, either percutaneously performed or by cutdown dissection.

### Axillary Artery Anatomy

Since in the majority of TAVI performed via trans-subclavian or TAx, the point of artery access is at the lateral border of the first rib^[9]^, in this paper, we will unify the terminology, referring to all cases as TAx route.

The axillary artery starts at the lateral border of the first rib and can be divided into three segments. The first segment is between the lateral border of the first rib and the medial border of the pectoralis minor; the second segment is behind the pectoralis minor; and the third segment is between the lateral border of the pectoralis minor and the inferior border of the teres major muscle^[9]^.

Previous studies have demonstrated that the mean vessel diameter is about 6.38 mm in the right axillary artery and 6.52 mm in the left one^[10]^, which means that it can accommodate sheaths with an outer diameter of up to 18 Fr. Moreover, this vessel is rarely affected by significant atherosclerosis (2.1% atherosclerotic disease in the axillary artery *vs*. 19.8% in the common femoral artery)^[10]^.

Comparing tissue elasticity, it is remarkable that the axillary artery has more elastic walls, while the femoral artery is more rigid due to muscular and fibrous components. This histological characteristic may have an impact on the efficacy of vascular closure devices used in axillary position^[9]^.

### Procedure Details

**Fig. 2:**
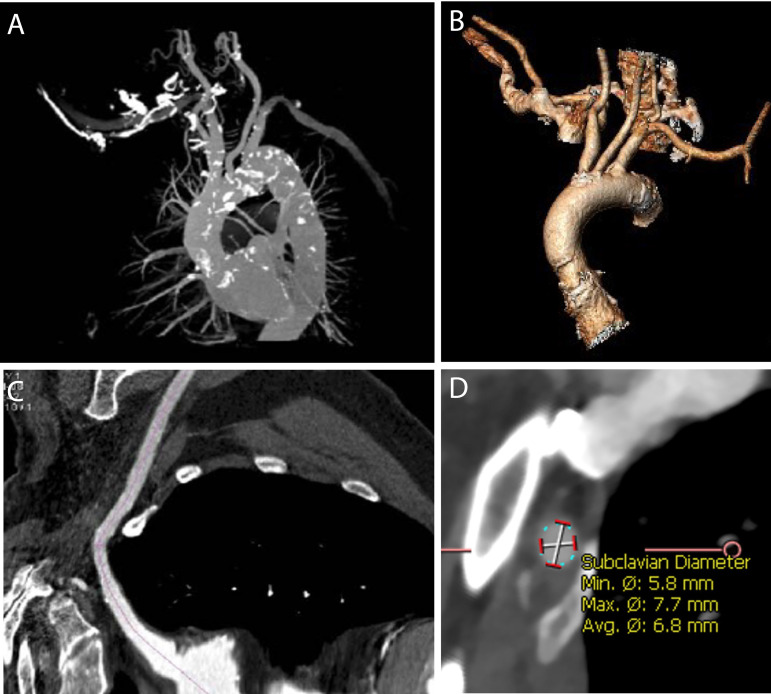
Transaxillary access evaluation. Axillary artery reconstruction in computed tomography (A-B). Vessel diameter, degree of tortuosity, relationship with side branches, and presence and extension of calcifications evaluation (B). Mean diameter suitable to transaxillary access (≥ 6 mm).

Preoperative high-resolution computed tomography scan aiming to screen the axillary artery suitability and plan the procedure is an essential step for a well-succeeded intervention. The critical points that should be evaluated are the vessel diameter, the degree of tortuosity, the relationship with side branches, and the presence and extension of calcifications (Figure 2).

According to Schäfer et al.^[9]^, three anatomical criteria must be taken into account when considering TAx approach^[11]^:

1. Subclavian or axillary artery diameter ≥ 6 mm;
2. Absence of heavy calcification, excessive kinking, or severe stenosis of the vessel to be accessed, unsuitable for balloon angioplasty;
3. In patients with a patent left internal mammary artery (LIMA) coronary bypass graft, a minimal vessel diameter of 7-8 mm and no significant atherosclerotic disease proximal to or at the ostium of the LIMA, in order to prevent myocardial ischemia.

The authors also emphasized that ipsilateral implanted pacemakers are not a contraindication for TAx approach.

Similar to these recommendations, in the recent Evolut Low-Risk trial, anatomical exclusion criteria were vessel mean diameter < 5.0 mm for Evolut R 23, 26, or 29; and < 5.5 mm for Evolut R 34 or Evolut PRO. If a patent LIMA graft was present, these values were modified to < 5.5 mm for Evolut R 23, 26, or 29; and < 6.0 mm for Corevalve 31, Evolut R 34, or Evolut PRO^[12]^.

In terms of technical aspects, the axillary artery can be reached by surgical cutdown or by totally percutaneous puncture. Regarding the site choice (right or left), it requires consideration of several factors. In general, the left axillary artery is preferred as it allows better coaxial orientation, decreases the chance of carotid compromise, and can be advantageous in right-handed patients. Other factors to be considered are the aortic annular angle and the takeoff angulation of the subclavian and innominate artery with the aortic arch. An angle > 30° between the annular plane and the right subclavian horizontal axis or > 70° between the annular plane and the left subclavian (*i.e*., "horizontal aorta") typically means a contraindication due to difficulties in achieving coaxiality. Type 1 arch (all three great vessels originating from the transverse arch) also represents a reason to avoid a right-sided approach, especially if the innominate artery arises distal on the arch. On the other hand, left-sided access may be challenging if the left subclavian artery is retroflexed towards the descending aorta, or with a steep subclavian to arch angulation (> 80°)^[13,14]^.

Despite the previous belief that the TAx approach requires general anesthesia, currently, about 46% of TAx-TAVI are done with local anesthesia and conscious sedation, especially during totally percutaneous access^[15]^. This approach might reduce procedural time, respiratory complications, postoperative delirium, and length of hospital stay^[11]^.

### Surgical Axillary Artery Approach

In this approach, the TAVI is directly delivered via subclavian/axillary artery. To perform the cutdown technique, the access is obtained by tissue dissection followed by placement of a double purse-string suture and direct cannulation; or by an arteriotomy, with attachment of a Dacron(r) graft^[16]^.

**Fig. 3:**
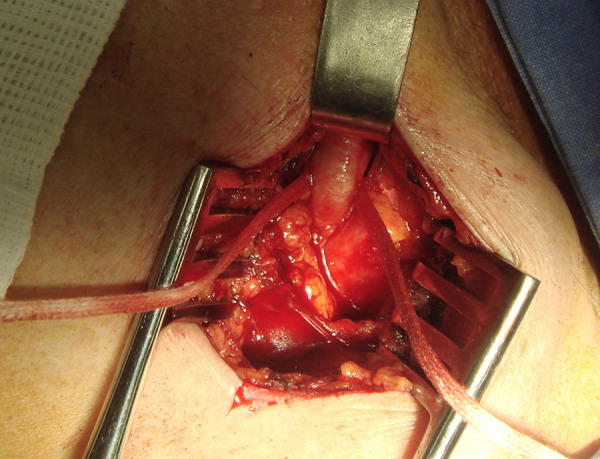
Axillary artery exposed and isolated with the use of two rubber vascular loops passed around its proximal and distal portions.

A direct cannulation approach begins with axillary artery exposition through a small, 3-5-cm transversal, infra-clavicular incision, performed 1 cm below and parallel to the clavicle. The pectoralis major muscle is slipped, the artery is exposed and encircled with soft rubber vessel loops (Figure 3). Attention is required to avoid damaging the brachial plexus. A double horizontal 5-0 polypropylene purse-string is performed, and the artery is punctured in the center of the purse-string. Following heparin administration (aiming to achieve an activated clotting time > 250 seconds), a 10 Fr sheath is placed over a soft, J-tip, 0.035 wire. The guidewire is switched for a stiff wire positioned in the apex of the left ventricle, using standard catheter-exchange techniques to cross the aortic valve. The 10 Fr introducer is exchanged by the delivery system. From this point, the procedure follows the same technique used for the TF route. At the end of the procedure, hemostasis is achieved by tightening the purse-string sutures, and the skin layers are closed in the usual fashion.

**Fig. 4:**
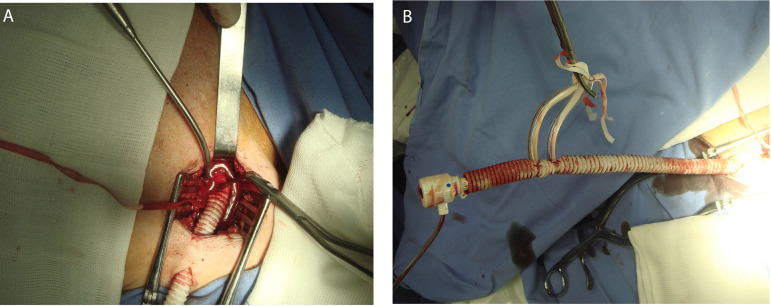
Transaxillary transcatheter aortic valve implantation performed through a Dacron tube graft insertion. Dacron tube graft anastomosed with the axillary artery (A). Sheath introduced through the Dacron tube graft (B).

In the Dacron graft approach^[16]^, a similar skin incision is carried out, the vessel is isolated with the use of two rubber vascular loops passed around its proximal and distal portions. A small incision in the anterior axillary wall is performed and a Dacron tube graft (usually 8 mm diameter x 15 cm length) is anastomosed using a 5-0 polypropylene (termino-lateral anastomosis). The Dacron graft is then clamped, and the proximal axillary artery is briefly unclamped to verify/optimize hemostasis. The tube is exteriorized through a second smaller and more lateral incision. The sheath is then introduced through the Dacron tube graft (Figure 4). At the end, the sheath is removed, and the graft is clamped with vascular surgical staple clips just above the anastomosis with the subclavian/axillary artery, avoiding any additional manipulation of the vessel. One of the Dacron graft approach main advantages is the cardiac surgeons' familiarity with this method since the axillary artery is frequently cannulated during aorta surgeries or ventricular assist device implantation. Besides, the incision is well tolerated by patients, associated with satisfying cosmetic results, and early mobilization^[17]^. On the other hand, a significant learning curve has been described, with technical proficiency beginning to develop at the 25^th^ case, and completing only after 50 cases^[18]^.

### Percutaneous Axillary Artery Approach

The potential benefits of percutaneous TAx access over other non-TF options are the maintenance of left ventricular integrity (in contrast to transapical) and the absence of chest opening (in comparison to direct aortic access), reducing procedural complexity and invasiveness^[4]^. Through this method, it is possible to offer a minimalist TAVI approach also to those patients with suboptimal femoral arteries^[11]^. A possible TAx limitation, however, is the assumed risk of vascular complications and bleeding^[4]^.

The ideal site for percutaneous axillary access is its first segment, just proximal to the thoracoacromial branch. This site is chosen based on invariant position and absence of overlying nerves or veins^[19]^. Besides this, some authors argue that, in this position, the axillary artery could be easily manually compressed against the second rib^[13]^.

A totally percutaneous axillary approach was described for the first time in 2012 by Schäfer et al.^[9]^ in a cohort of 24 high-risk patients (mean logistic European System for Cardiac Operative Risk Evaluation I [EuroScore I] score 35.3±22.8%; Society of Thoracic Surgeons [STS] score > 10%). The authors reported no major adverse cardiac or cerebrovascular events, and no major vascular complications. Device success was obtained in 95.8%, and the 30-day mortality was 8.3%. A particularly interesting observation was that, when the ProStar system was used as vascular closure device, 29.2% of patients (n=7) needed a vascular stent placement because of persistent bleeding or closure failure, whereas when the Perclose ProGlide system was used, no complications were observed.

The expanded experience of the so-called "Hamburg Sankt Georg Approach", comprising 100 consecutive cases, was published in 2017, using the left artery in 85% and the right artery in 15% of cases. Device success was achieved in 95%, there was one procedural death (annular rupture), one peri-procedural transient ischemic attack, and the one-year mortality was 14.8%. Vascular stent placement was required in 11 patients, mainly in the early phase (seven reported in the initial study phase and four in the extended follow-up). These data made authors conclude that a learning curve clearly exists, and the complication rate tends to decrease with growing experience^[4]^.

**Fig. 5:**
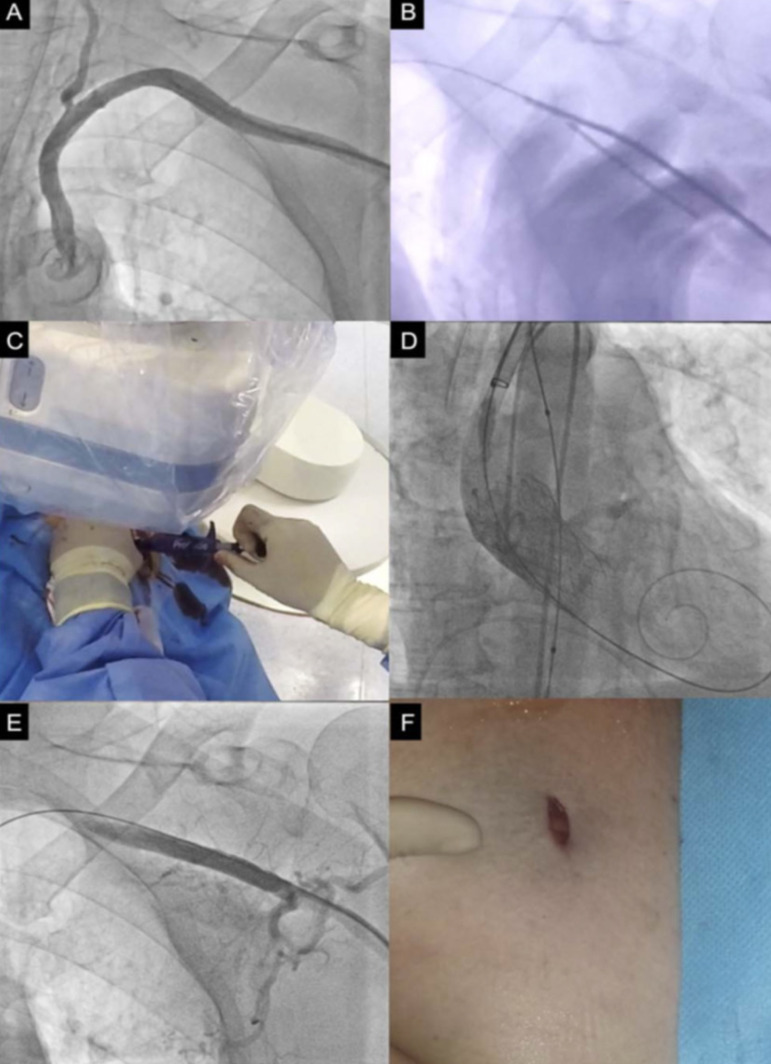
Fluoroscopic and angiography-guided axillary artery approach. Contrast injection aiming to mark the axillary artery trajectory (A). Axillary artery puncture using as reference fluoroscopic landmarks and a guidewire inserted through the radial artery (B). Perclose technique using the Perclose/ProGlide Suture-Mediated Closure System (C). Transcatheter aortic valve implantation deployment (D). Final angiographic control (E). Final skin incision aspect (F).

**Fig. 6:**
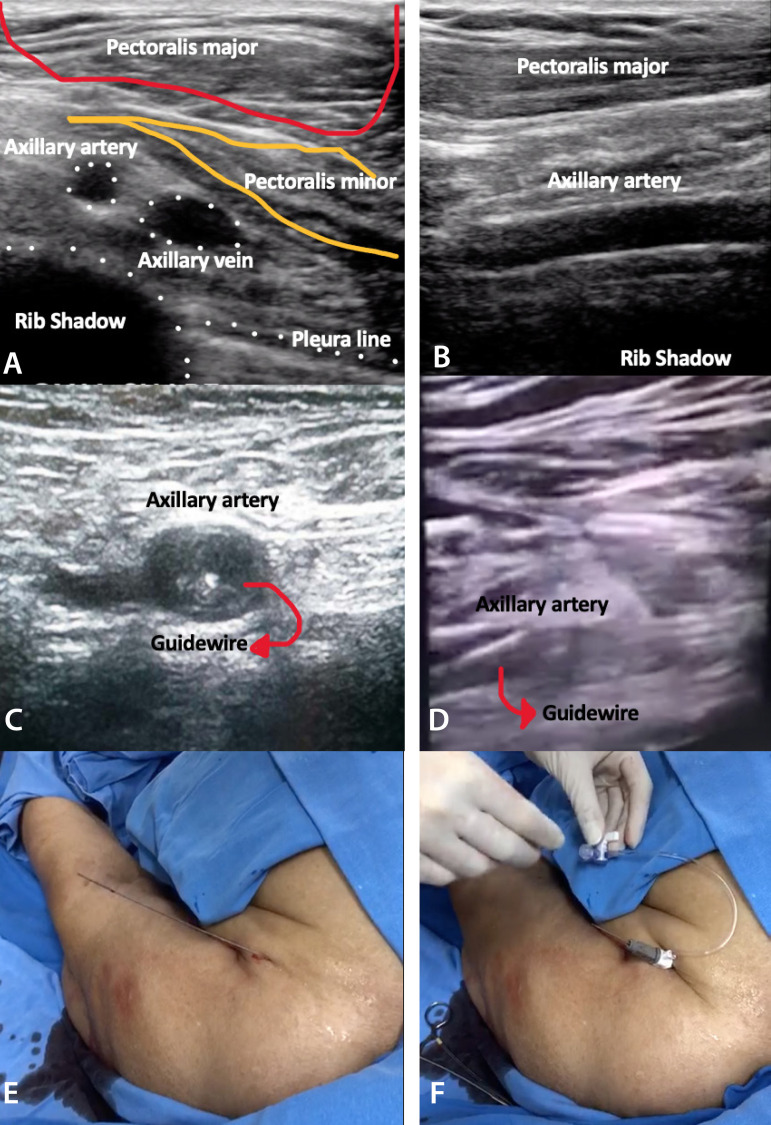
Ultrasound-guided axillary artery puncture. Ultrasound short (A) and long-axis (B) axillary artery evaluation. Out-of-plane (shot-axis) (C) and in-plane (longitudinal-axis) axillary artery puncture (D). Guidewire (E) and short sheath insertion (F).

Regarding procedural details, all axillary punctures were performed guided by fluoroscopy, using a regular J-wire as a landmark (an example of this approach is showed in Figure 5). The mean minimum vessel diameter was 6.85±0.17 mm, and nine patients presented moderate access vessel calcification. The puncture was performed in the proximal location of the first segment, at a distance of 1.2±0.9 cm to the lateral border of the rib, to avoid pneumothorax and to have the possibility of manual artery compression^[4,9]^. Nowadays, ultrasound-guided axillary artery puncture has been used as an alternative access to fluoroscopy or angiography-guided punctures (Figure 6).

## DISCUSSION

Since the first TAx-TAVI case performed by Ruge H. in 2008^[20]^, several technical improvements and increased operator familiarity with the method contributed to make this approach the second route of choice in many TAVI centers. In this pioneer report, a patient with aortoiliac occlusive disease and concomitant left subclavian arterial stenosis received a TAVI through the right axillary artery. One year later, the left axillary artery access was described^[21]^ in a woman with severe AS and small iliofemoral arteries. Following these pioneer reports, many series of cases started to describe the outcomes associated with the TAx route.

One of the first analysis from the Italian national registry showed that, even when performed in higher-risk patients, TAx-TAVI presented similar outcomes compared to the TF route in terms of procedural success (100% *vs*. 98.4%; *P*=0.62), intraprocedural mortality (0% *vs*. 0.9%; *P*=1.00), and valve-related adverse events (13.6% *vs*. 13.9%; *P*=0.79)^[22]^. When propensity score matching was used, the TAx approach showed an equivalent two-year survival (74.0±4.0% in TAx *vs*. 73.7±3.9% in TF; *P*=0.78), and lower rates of acute kidney injury (4.3% *vs*. 9.9%; *P*=0.02), minor vascular complications (2.1% *vs*. 11.3%; *P*=0.003), and major bleeding due to vascular complication (0.7% *vs*. 4.3%; *P*=0.05)^[23]^.

Amat-Santos also suggested that, despite a higher logistic EuroScore (23.7±1.92 *vs*. 21.17±3.51; *P*=0.04) and more prevalent coronary and peripheral artery disease, TAx access was performed with a similar 30-day stroke rate, need for new pacemaker implantation, major vascular complications, and acute kidney injury requiring dialysis when compared to the TF approach^[24]^.

In addition to the aforementioned data, no differences between TF and TAx approaches in terms of procedural success rate (97.9% TAx *vs*. 95-100% TF), neurological events (2.1% TAx *vs*. 1.4-6.7% TF), new pacemaker implant (24.7% TAx *vs*. 3.4-34.1% TF)^[25]^, procedural time, procedural success^[8]^, paravalvular leak, conversion to open-heart surgery, and hospital length of stay^[26]^ were also suggested.

Comparing the most common used routes, a meta-analysis published in 2015 encompassing 17,020 patients (11,079 TF and 5,941 non-TF [5,119 transapical, 514 TAx, 305 transaortic, and three transcarotid)] showed a lower 30-day (4.7% TF *vs*. 8.1% non-TF; *P*<0.01) and one-year mortality rate (16.4% TF *vs*. 24.8% non-TF; *P*<0.01) in patients who underwent a TAVI procedure through TF access. TF access was, however, associated with higher incidence of vascular complications (odds ratio [OR] 2.1; 95% confidence interval [CI] 1.48-2.99) and similar incidence of bleeding (OR 1.01; 95% CI 0.81-1.27) and cerebrovascular events (1.6% *vs*. 2.1%; *P*=0.31) compared to TAx access^[27]^.

Trend analysis revealed that, among 3,628 patients from the STS/American College of Cardiology Transcatheter Valve Therapy registry who received a balloon-expandable SAPIEN 3 prosthesis (Edwards Lifesciences LLC, Irvine, California, United States of America) via non-TF access, the axillary artery was used in 20.2% in the third quarter of 2015, and in 49.0% in the fourth quarter of 2017 (*P*<0.001). This growth was accompanied by a concomitant decrease in transapical and transaortic use (from 61.9% in the third quarter of 2015 to 35.3% in the fourth quarter of 2017; *P*<0.001). Comparing both methods by propensity score matching, the TAx route was associated with lower 30-day mortality (5.3% *vs*. 8.4%; *P*<0.01), new-onset atrial fibrillation (2% *vs*. 13%; *P*<0.001), new dialysis requirement (0.7% *vs*. 2.5%; *P*=0.001), rehospitalization (11.6% *vs*. 15.1%; *P*=0.03), and shorter intensive care unit (26.3 hours *vs*. 47 hours; *P*<0.001), and hospital length of stay (three days *vs*. six days; *P*<0.001), but a higher stroke rate (6.3% *vs*. 3.1%; *P*<0.05)^[28]^.

A sub-analysis from this same study showed no significant difference in adverse procedural outcomes when percutaneous axillary artery access was compared to the surgical one. Despite slightly shorter fluoroscopy times and longer intensive care unit length of stay in the surgical group, there were no differences in 30-day mortality, stroke, new-onset atrial fibrillation, readmission, new requirement for dialysis, new pacemaker implantation, life-threatening bleeding, and major vascular complication ^[28]^.

This same tendency favoring the TAx approach as the second route across the years was seen with the self-expanding Evolut R system (Medtronic, Minneapolis, Minnesota, United States of America), with a ratio of 3.3:1 between TAx and direct aortic route^[29]^.

## CONCLUSION

With transcatheter valve technology expanding its indications for low-risk patients, the number of TAVI-eligible patients will globally grow, requiring a better understanding about the second-best access choice.

Although no direct comparison from prospective, randomized trials is available, the TAx approach has emerged as a feasible alternative, with similar results to the TF approach, and better than other non-TF approaches. Vascular access route and technical aspects should be a patient-centered decision rather than an operator-driven preference.

**Table:** 

Authors' roles & responsibilities	
APT	Substantial contributions to the conception or design of the work; or the acquisition, analysis, or interpretation of data for the work; drafting the work or revising it critically for important intellectual content; agreement to be accountable for all aspects of the work in ensuring that questions related to the accuracy or integrity of any part of the work are appropriately investigated and resolved; final approval of the version to be published
RPS	Substantial contributions to the conception or design of the work; or the acquisition, analysis, or interpretation of data for the work; drafting the work or revising it critically for important intellectual content; agreement to be accountable for all aspects of the work in ensuring that questions related to the accuracy or integrity of any part of the work are appropriately investigated and resolved; final approval of the version to be published
EF	Substantial contributions to the conception or design of the work; or the acquisition, analysis, or interpretation of data for the work; drafting the work or revising it critically for important intellectual content; agreement to be accountable for all aspects of the work in ensuring that questions related to the accuracy or integrity of any part of the work are appropriately investigated and resolved; final approval of the version to be published
MT	Substantial contributions to the conception or design of the work; or the acquisition, analysis, or interpretation of data for the work; drafting the work or revising it critically for important intellectual content; agreement to be accountable for all aspects of the work in ensuring that questions related to the accuracy or integrity of any part of the work are appropriately investigated and resolved; final approval of the version to be published
EKS	Substantial contributions to the conception or design of the work; or the acquisition, analysis, or interpretation of data for the work; drafting the work or revising it critically for important intellectual content; agreement to be accountable for all aspects of the work in ensuring that questions related to the accuracy or integrity of any part of the work are appropriately investigated and resolved; final approval of the version to be published
